# Brain-Targeted Reactive Oxygen Species in Hypertension: Unveiling Subcellular Dynamics, Immune Cross-Talk, and Novel Therapeutic Pathways

**DOI:** 10.3390/antiox14040408

**Published:** 2025-03-28

**Authors:** Renjun Wang, Min Wang, Dongshu Du, Zhiying Shan, Lanrong Bi, Qing-Hui Chen

**Affiliations:** 1 Department of Biotechnology, School of Life Science, Jilin Normal University, Siping 136000, China; renjunwang211@163.com (R.W.); 17319734210@163.com (M.W.); 2 Department of Kinesiology and Integrative Physiology, Michigan Technological University, Houghton, MI 49931-1200, USA; zhiyings@mtu.edu; 3 School of Life Sciences, Shanghai University, Shanghai 200444, China; dsdu@shu.edu.cn; 4 Department of Chemistry, Michigan Technological University, Houghton, MI 49931-1200, USA

**Keywords:** oxidative stress, reactive oxygen species, antioxidants, cardiovascular diseases, hypertension

## Abstract

Hypertension (HTN) is a complex disease with significant global health implications, driven by neural and oxidative mechanisms. Reactive oxygen species (ROS), once considered mere metabolic byproducts, are now recognized as one of the key contributors to dysfunction of the autonomic nerve system, which involves the onset and progression of HTN. This review highlights the dynamic roles of ROS in neuronal signaling, subcellular compartmentalization, and brain–immune interactions, focusing on their impacts on synaptic remodeling, neuroinflammation, and epigenetic modifications within key autonomic regions such as the paraventricular nucleus and rostral ventrolateral medulla. We discuss novel ROS sources, including microglia-derived and endoplasmic reticulum stress-related ROS, and their contributions to HTN. Subcellular dynamics, such as ROS signaling at mitochondria-associated membranes and neuronal microdomains, are explored as activators of the sympathetic nerve system. Emerging evidence has linked ROS to epigenetic regulation, including histone modifications and non-coding RNA expression, with sex-specific differences offering insights for the development of personalized therapies. Innovative therapeutic strategies targeting ROS involve precision delivery systems, subcellular modulators, and circadian-optimized antioxidants. We propose several priorities for future research, including the real-time imaging of brain ROS, translating preclinical findings into clinical applications, and leveraging precision medicine to develop tailored interventions based on ROS activity and genetic predisposition. Through emphasizing the spatial and temporal complexity of ROS in HTN, this review identifies novel therapeutic opportunities and establishes a foundation for targeted treatments to address this health challenge.

## 1. Introduction

### 1.1. Hypertension as a Multi-Factorial Disease: Beyond Traditional Perspectives

Hypertension (HTN) is associated with hemodynamic, genetic, and environmental factors. However, emerging evidence has highlighted the critical roles of the nervous system and oxidative stress, including an increase in reactive oxygen species (ROS) in its pathogenesis [1]. The abnormal activation of the sympathetic nervous system (SNS) increases peripheral vascular resistance and cardiac output, which significantly elevates blood pressure [2]. Concurrently, ROS exacerbates HTN by damaging the vascular endothelium and impairing nitric oxide production, leading to vasoconstriction and vascular stiffness [3]. The interplay between neural factors and ROS underscores the complexity of HTN and presents novel avenues for therapeutic strategies.

While ROS is widely recognized as a contributor to HTN, most research has focused on systemic ROS, leaving the role of brain-specific ROS underexplored. The brain regulates blood pressure through mechanisms such as the autonomic nervous system and the hypothalamic–pituitary–adrenal (HPA) axis [4]. Nevertheless, the impact of ROS within the brain remains poorly understood. Patocka et al. suggested that brain-specific ROS may have a profound effect on neurotransmission and vascular tone, contributing to autonomic nervous system dysfunction and potentially surpassing the influence of peripheral ROS [5]. Investigating the link between brain ROS and HTN could address this research gap and establish the brain’s central role in the pathogenesis of the disease.

### 1.2. The Role of Brain ROS in Understanding the Pathogenesis of Hypertension

ROS in the brain play a crucial role in regulating arterial blood pressure (ABP) through their influence on the autonomic nervous system, particularly the balance between sympathetic and parasympathetic activity [6]. Liu et al. have demonstrated that the endoplasmic reticulum (ER)–mitochondrial association mediated by PDZD8 impacts ROS levels and blood pressure regulation in the rostral ventrolateral medulla (RVLM) of Sprague Dawley (SD) rats [7]. Similarly, Duan et al. investigated how circadian rhythm disruptions in rats increase ROS in the RVLM, leading to sympathetic overactivity and subsequent HTN, as well as cardiovascular complications [8]. Furthermore, Xi highlighted the impact of ROS on salt–water balance and vasoconstriction, mediated through brain regions such as the hypothalamus and cortex [9]. These findings underscore that studying brain ROS offers valuable insights into the pathogenesis of HTN and allows for the identification of potential therapeutic targets for intervention.

### 1.3. Scope of the Review

This review focuses on the emerging roles of ROS as critical modulators in the pathogenesis of HTN. It synthesizes the recent advances in understanding the spatial and temporal dynamics of ROS within the brain’s autonomic regions, particularly the paraventricular nucleus of hypothalamus (PVN) and RVLM. The review explores the ways in which ROS are involved in neuronal signaling, subcellular compartmentalization, and cross-talk with immune responses, highlighting their contributions to synaptic remodeling, neuroinflammation, and epigenetic modifications.

The scope of the review extends to discuss novel sources of ROS, such as microglia-derived and endoplasmic reticulum-associated ROS, and their role in amplifying sympathetic outflow. Additionally, subcellular mechanisms such as ROS signaling at mitochondria-associated membranes and neuronal microdomains are examined. The review delves into ROS-driven epigenetic changes, including histone modifications and non-coding RNA expression, as well as sex-specific differences in ROS responses and their implications for precision medicine.

Therapeutic approaches targeting ROS are analyzed, including precision delivery systems, subcellular ROS modulators, and circadian-aligned antioxidant strategies. The review concludes by identifying key gaps in the current knowledge and proposing future research directions, such as advanced imaging techniques, translational studies, and the development of individualized therapies. Through this comprehensive synthesis, the review aims to provide a deeper understanding of the role of ROS in HTN and outline innovative strategies for intervention. In addition, this review also aims to analyze the complex mechanisms underlying the physiological processes and pathological changes in brain ROS. Studying the generation, clearance, signal transduction mechanisms of ROS is beneficial for providing disease prevention strategies and developing new therapeutic drugs.

## 2. Advancing the Understanding of ROS

### 2.1. Novel Sources and Contexts of ROS Production in Hypertension

#### 2.1.1. Underexplored Sources of ROS: ER Stress and Microglia-Derived ROS

Emerging research has identified several underexplored sources of ROS that contribute to the development of HTN, beyond their well-known mitochondrial and vascular origins. These include ROS derived from ER stress, Golgi apparatus stress, astrocytes, and microglia [10]. ER stress triggers ROS production through the activation of the unfolded protein response pathway. The generated ROS not only disrupt protein folding and degradation but also impair endothelial function and promote smooth muscle contraction, which contribute to the increase in ABP [11]. Additionally, microglia—as central nervous system immune cells—produce ROS that influence neuronal activity, which could underlie a novel mechanism for the pathophysiology of HTN [12].

#### 2.1.2. Role of Aldosterone-Induced ROS in Hypertension

Aldosterone plays both a physiological role and a pathophysiological process in the central nervous system. Increases in the level of aldosterone can activate the brain renin–angiotensin system, which would inhibit the activities of superoxide dismutase (SOD) and glutathione peroxidase (GPx). These changes are able to increase the products of lipid peroxidation, including malondialdehyde (MDA), thus increasing ROS and sympathetic outflow [13,14]. Moreover, it has been reported that aldosterone is able to downregulate the activity of Nrf2 and inhibit the antioxidant effect, which induce ROS and cause vascular dysfunction [15], leading to the development of HTN and heart failure. Consequently, the hypothalamus–pituitary–adrenal (HPA) axis and the renin–angiotensin–aldosterone system both play a role in the development of HTN and heart failure [16,17,18,19]. Understanding the mechanisms of aldosterone-induced ROS, which are related to the pathophysiological process of HTN and heart failure, will provide a new target for the subsequent clinical treatment of these cardiovascular diseases.

#### 2.1.3. ROS Generation in Normotensive vs. Hypertensive States

The mechanisms and effects of ROS production are different between normotensive and hypertensive conditions. In normotensive states, ROS production and clearance are balanced, exerting minimal influence on ABP. In contrast, the ROS activity is enhanced in hypertensive states, which is characterized by increased ROS levels in the mitochondria, endothelial cells, and immune cells [20,21]. The excessive ROS observed in HTN contribute to endothelial dysfunction, the activation of SNS, and systemic inflammation, all of which lead to an increase in ABP [22]. Moreover, elevated ROS levels promote smooth muscle cell proliferation and contraction, exacerbating vascular sclerosis and accelerating the progression of HTN [23]. These findings underscore the pivotal role of ROS in the pathogenesis of HTN.

### 2.2. Dynamic Role of ROS in Brain Plasticity and Hypertension Progression

ROS influences synaptic plasticity, particularly within autonomic regions such as the hypothalamus and brainstem. By altering the structure and function of neural synapses, ROS interfere with neuronal signal transmission, affecting the balance between the sympathetic and parasympathetic nervous systems. Increased ROS levels in the brain enhance sympathetic activity while suppressing parasympathetic activity, leading to peripheral vascular constriction and the development of HTN [24,25]. Studies have shown that ROS act on neural circuits within the hypothalamus, brainstem, and cerebral cortex to regulate the salt–water balance and vascular contraction, thereby enhancing sympathetic activity and raising ABP [25]. The inhibition of ROS production has been demonstrated to reduce SNS activation and effectively control blood pressure, further emphasizing the critical role of brain-specific ROS in HTN [26]. These insights highlight the decisive influence of ROS on the onset and progression of HTN through their effects on neurocircuitry.

## 3. Emerging Insights into Subcellular ROS Localization in Hypertension

### 3.1. Compartmentalized ROS Production in Subcellular Organelles

#### 3.1.1. The Role of Endoplasmic Reticulum–Mitochondrial Contact Sites in ROS Generation

**Figure 1:**
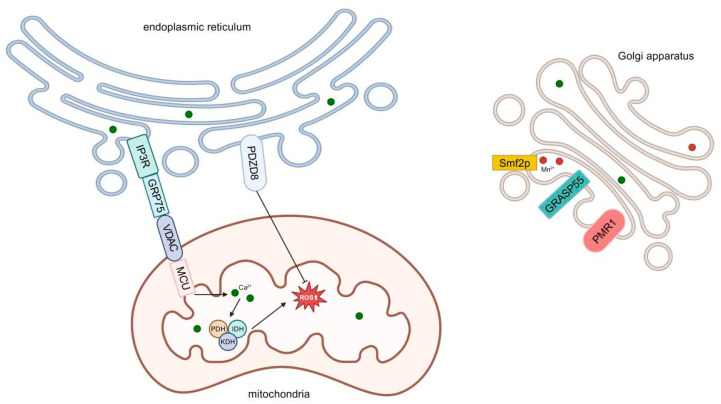
Certain proteins in the mitochondria-associated membrane (MAM) regions are involved in the regulation of calcium homeostasis. Stored calcium ions in the endoplasmic reticulum are released by inositol 1,4,5-trisphosphate receptor (IP3R) and transferred to the mitochondria through the mitochondrial protein voltage-dependent anion channel (VDAC) and mitochondrial calcium uniporter (MCU) channels; glucose-regulated protein 75 (GRP 75) is also involved in this process. Calcium ions entering the mitochondria activate dehydrogenases such as pyruvate dehydrogenase (PDH), isocitrate dehydrogenase (IDH), and α-ketoglutarate dehydrogenase (KDH), increasing the electron donors of electron transport chain (ETC) and promoting the generation of ROS. Proline and zinc finger domain containing 8 (PDZD 8) plays a protective role in endoplasmic reticulum (ER) stress and suppresses ROS generation. The Golgi reassembly stacking protein of 55 (GRASP55) plays an important role in Golgi stacking and pulls the Golgi body to the mitochondria. Loss of the Golgi plasma membrane calcium ATPase 1 (PMR 1) sensitizes the Golgi to ROS, thereby inhibiting ROS production. The manganese transporter 2 protein (Smf2p) can regulate the flow of manganese and calcium ions within the Golgi body, affecting the level of ROS.

Mitochondria-associated membranes (MAMs) are specialized regions within cells that play a critical role in ROS generation. These regions are formed by the close proximity of the ER and mitochondria, typically separated by a distance of 10–20 nm. MAMs are stabilized by protein complexes, including the inositol 1,4,5-trisphosphate receptor (IP3R) and the voltage-dependent anion channel (VDAC), which facilitate calcium ion (Ca^2+^) signaling and metabolite exchange between the ER and mitochondria. These interactions establish the structural basis for ROS generation (Figure 1).

In response to ER signals, IP3R releases Ca^2+^ into the cytoplasm, a portion of which is subsequently taken up by mitochondria. Elevated mitochondrial Ca^2+^ concentrations activate dehydrogenases such as pyruvate dehydrogenase (PDH), isocitrate dehydrogenase (IDH), and α-ketoglutarate dehydrogenase (KDH). This activation accelerates the tricarboxylic acid (TCA) cycle, increasing the availability of electron donors for the mitochondrial electron transport chain (ETC); the enhanced ETC activity drives ROS production.

Under normal conditions, MAMs maintain the functionality of the ETC, promoting efficient electron transport and maintaining ROS levels within physiological limits. However, cellular stress can disrupt the structure or function of MAMs, leading to ETC disassembly, impaired electron transport, and increased electron leakage. These alterations result in excessive ROS generation, contributing to cellular dysfunction and damage.

#### 3.1.2. ROS Communication Between Organelles and Its Impact on Neuronal Function

ROS generation and responses occur in a dynamic interplay among organelles. Mitochondrial ROS can diffuse to the ER, altering its redox environment and disrupting protein folding. Conversely, ER stress-induced ROS can impair mitochondrial respiratory function and membrane potential, further amplifying mitochondrial ROS production. This inter-organelle ROS communication significantly affects neuronal functions.

Excessive ROS disrupt the synthesis and release of neurotransmitters in neurons [27]. For example, elevated ROS levels in presynaptic neurons can alter the activity of enzymes involved in synthesizing neurotransmitters such as glutamate and γ-aminobutyric acid (GABA) [28]. Additionally, ROS can impair synaptic plasticity mechanisms such as long-term potentiation and long-term depression, leading to deficits in learning, memory, and neuronal survival [29].

### 3.2. Role of Microdomains in Neuronal ROS Signaling

ROS hotspots form on neuronal membranes due to the activities of specific enzymes and receptors. NADPH oxidases (NOXs) are widely distributed on neuronal membranes and play a central role in ROS generation. Under stimulation, NOXs transfer electrons from intracellular NADPH to oxygen, producing superoxide anions (O_2_⁻·), thus initiating ROS hotspot formation [30]. Similarly, certain cytochrome P450 enzymes generate ROS during substrate metabolism, contributing to localized ROS accumulation [31].

Neuronal membrane receptors also contribute to ROS hotspot formation; for example, the angiotensin II receptor (AT1R), upon binding to angiotensin II, activates phospholipase C (PLC). PLC hydrolyzes phosphatidylinositol bisphosphate (PIP_2_), producing inositol trisphosphate (IP_3_) and diacylglycerol (DAG). IP₃ induces Ca^2+^ release from intracellular stores, and the resulting Ca^2+^–calmodulin complex activates NOX, generating significant ROS near the receptor [32]. Similarly, G protein-coupled receptors (GPCRs), such as G protein-coupled receptor 17, mediate the production of ROS through distinct signaling pathways [33].

The non-uniform distribution of these receptors creates localized ROS generation, amplifying specific neuronal signals and contributing to the formation of microdomain-specific ROS hotspots, which play a critical role in the amplification of signals in SNS.

## 4. Cross-Talk Between ROS and Brain–Immune Interactions

### 4.1. ROS-Driven Neuroinflammation in Autonomic Regions of Brain

#### 4.1.1. Mechanisms Linking ROS with Microglial Activation and Cytokine Release in PVN, RVLM, and Nucleus Tractus Solitarii (NTS)

ROS directly interacts with proteins and lipids on the surface of microglia, inducing oxidative modifications. For instance, ROS can oxidize receptor proteins on microglial surfaces, altering their conformation and activating previously dormant receptors. Once inside microglia, ROS stimulate multiple intracellular signaling pathways, with the mitogen-activated protein kinase (MAPK) pathway playing a pivotal role. Specifically, ROS activate members of the MAPK family, including extracellular signal-regulated kinase (ERK), p38 MAPK, and c-Jun N-terminal kinase (JNK). These kinases, in turn, phosphorylate downstream target proteins such as transcription factors including nuclear factor-κB (NF-κB) [34].

Activated NF-κB translocates into the nucleus and promotes the expression of genes associated with microglial activation, such as inducible nitric oxide synthase (iNOS) [35] and cyclooxygenase-2 (COX-2). The resulting gene products further amplify the neuroinflammatory response by releasing additional ROS and pro-inflammatory cytokines, creating a self-sustaining feedback loop [36,37].

**Figure 2:**
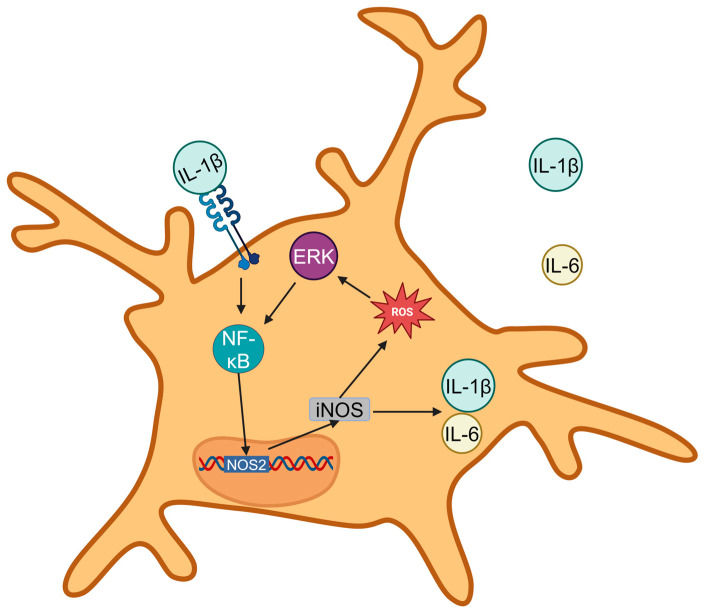
ROS in microglia activate extracellular signal-regulated kinase (ERK), ERK phosphorylates nuclear factor kappa B (NF-κB), and NF-κB enters the nucleus to initiate *nitric oxide synthase 2* (*NOS2)* expression and promote the release of cytokines interleukin-1β (IL-1β), interleukin-6 (IL-6), and ROS. The extracellular pro-inflammatory factor IL-1β binds to interleukin-1 receptor (IL-1R) to activate NF-κB, creating a vicious cycle.

Microglia, once activated, release a variety of cytokines. In the PVN, RVLM, and NTS, the primary pro-inflammatory cytokines are tumor necrosis factor-α (TNF-α), interleukin-1β (IL-1β), and interleukin-6 (IL-6) [38,39]. These cytokines are released through both classical and non-classical secretion pathways, perpetuating the neuroinflammatory cycle (Figure 2).

#### 4.1.2. Feedback Loops Sustaining ROS and Inflammatory Signaling in Hypertension

In the pathogenesis of HTN, elevated ROS levels in autonomic brain regions, including the PVN, RVLM, and NTS, are closely linked to neuroinflammation [6,40]. ROS activate microglia through various mechanisms, such as directly oxidizing surface receptors, which shifts them into an activated state. Activated microglia then release pro-inflammatory cytokines, including TNF-α and IL-1β.

Within the PVN, heightened ROS levels activate intracellular signaling pathways in microglia, leading to the release of TNF-α. TNF-α subsequently stimulates neurons to produce more ROS, establishing a vicious cycle [41]. Similar processes occur in the RVLM and NTS, where ROS-driven cytokine release disrupts neuronal function and influences systemic blood pressure through neuroendocrine and autonomic pathways [12].

Inflammatory cytokines perpetuate microglial activation via autocrine and paracrine signaling; for example, IL-1β binds to IL-1 receptors on microglia, activating intracellular pathways such as MAPK and NF-κB, which maintain microglia in a persistently activated state. This chronic activation leads to continuous ROS and cytokine production, forming a central feedback loop that collaborates with peripheral ROS and inflammatory signals [40]. Together, these mechanisms drive the development and persistence of HTN.

### 4.2. Astrocytic ROS: A Novel Contributor to Autonomic Dysregulation

#### 4.2.1. Impact of Astrocytic ROS on Neuronal Excitability and Neurotransmitter Balance

In terms of their impact on neuronal excitability, astrocytic ROS mainly play a role by regulating ion channels and neurotransmitter receptors [42]. From the perspective of ion channels, ROS can modify multiple ion channels on the neuronal membrane; for example, the inwardly rectifying potassium ion channel (Kir) is of great significance for maintaining the resting membrane potential of neurons. ROS generated by astrocytes can oxidatively modify the Kir channel thus reducing its conductance [43]. As a result, the neuronal membrane potential depolarizes, shifting closer to the threshold for generating an action potential, and neuronal excitability consequently increases. Voltage-gated sodium ion channels are also affected by ROS. Their gating kinetic characteristics are altered, making them more likely to open during the depolarization process, increasing the influx of sodium ions and further enhancing neuronal excitability. In terms of neurotransmitter balance, astrocytes also play a crucial role. In the glutamate–glutamine cycle, astrocytes convert glutamate into glutamine through glutamine synthetase, providing precursors for neurons to synthesize glutamate [44]. However, ROS can inhibit the activity of glutamine synthetase, reducing the production of glutamine, which in turn leads to a decrease in the amount of glutamate synthesized by neurons and disrupts the balance between excitatory and inhibitory neurotransmitters. Regarding the synthesis of GABA—which is produced in neurons through the catalysis of glutamate by glutamate decarboxylase (GAD)—astrocytic ROS can indirectly affect the activity of GAD by changing the intracellular redox environment of neurons. When the ROS level increases, the GAD activity decreases, the synthesis of GABA is reduced, and the neurotransmitter balance tends to be excitatory [45].

#### 4.2.2. Role of Astrocytes in Amplifying the Hypertensive Response

Astrocytes play a role in amplifying the hypertensive response through their interactions with neurons and blood vessels [46]. ROS produced by astrocytes can affect the tension of cerebral vessels, leading to abnormal vasoconstriction or dilation [47]. Additionally, the signal transmission between astrocytes and neurons changes during HTN. Some signal molecules released by astrocytes can promote the abnormal firing of neurons under the influence of ROS, which further affects sympathetic output and causes an increase in ABP. Studies have shown that inhibiting the production of ROS in astrocytes can partially relieve the symptoms in animal models of HTN. Complex interactions occur between astrocytes and blood vessels [48]. On one hand, astrocytes can release some vasoactive substances, such as prostaglandins. During HTN, prostaglandins released by astrocytes after being stimulated may affect the contraction and dilation of vascular smooth muscle; for example, some prostaglandins may cause the contraction of vascular smooth muscle, reducing the diameter of blood vessels and increasing blood pressure [49,50]. On the other hand, astrocyte end feet closely surround cerebral blood vessels and form part of the blood–brain barrier (BBB). Changes in the functions of astrocytes caused by HTN may disrupt the integrity of the BBB, making it easier for some substances in the blood to enter the brain, affecting the regulation of the cardiovascular function and contributing to the development of hypertension.

## 5. ROS and Epigenetic Regulation in Brain Hypertension

### 5.1. Histone Modifications and DNA Methylation Regulated by ROS in Hypertensive States

ROS can directly oxidize histones and influence the activity of histone acetyltransferases (HATs) and histone deacetylases (HDACs). Increased ROS levels, as observed in hypertensive states, can suppress HAT activity or enhance HDAC activity thus reducing histone acetylation. For instance, in animal models of HTN, ROS have been demonstrated to significantly decrease histone acetylation in the brain, cardiovascular tissues, and other related cells, when compared to the normal physiological conditions [51,52,53].

**Figure 3:**
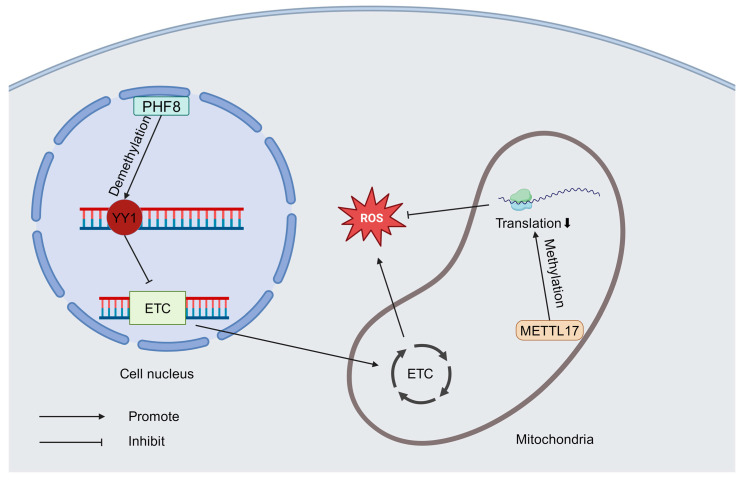
PHD finger protein 8 (PHF8) demethylates the *Yin Yang 1* (*YY1)* gene, inhibits the expression of ETC-related genes, and suppresses the ROS level. The mitochondrial protein methyltransferase like 17 (METTL17) methylates mitochondrial RNA, leading to inhibition of mitochondrial protein expression, which suppresses ROS levels.

DNA methylation, which is facilitated by DNA methyltransferases (DNMTs), is another epigenetic mechanism affected by ROS. Elevated ROS levels in HTN can oxidize critical amino acid residues in DNMTs—particularly cysteine residues—altering their enzymatic activity. Studies have shown that DNMT activity is significantly reduced in hypertensive models, leading to changes in DNA methylation patterns [54,55,56]. Additionally, ROS modulate demethylase activity; for instance, the demethylase PHF8 can decrease the methylation level of *YY1*, thereby downregulating the expression of ETC genes and reducing ROS levels [57]. Similarly, the mitochondrial protein METTL17 methylates mitochondrial RNA, affecting mitochondrial protein translation and function, ultimately inhibiting ROS production [58] (Figure 3).

ROS-induced epigenetic changes in HTN can have transgenerational effects, mediated through the transmission of abnormal epigenetic markers via germ cells. During gametogenesis, these altered markers may persist and be passed to offspring upon fertilization, influencing gene expression patterns and physiological functions in subsequent generations [59]. For example, parental HTN can result in altered DNA methylation patterns in sperm, leading to abnormal methylation in specific gene promoter regions. These changes can disrupt gene expression during embryonic development and affect the growth of the offspring [60].

### 5.2. Non-Coding RNAs as Mediators of ROS Signaling in Hypertension

HTN alters the expression of certain microRNAs (miRNAs), affecting the regulation of ROS-producing enzymes. miRNAs regulate gene expression at the post-transcriptional level and are integral to cellular processes such as ROS and apoptosis. Specific miRNAs are expressed in response to ROS to mediate ROS, while ROS can up-regulate the miRNAs that mitigate oxidative damage [40,61].

Long non-coding RNAs (lncRNAs) also play significant roles in ROS regulation and epigenetic processes. Some lncRNAs influence ROS production through modulating the expression and activity of NADPH oxidase. For instance, certain lncRNAs inhibit the expression of NADPH oxidase subunits, thereby reducing superoxide anion generation. Additionally, lncRNAs regulate mitochondrial function, including mitochondrial biogenesis, in order to decrease mitochondrial ROS production [62].

Non-coding RNA plays a key role in the process of oxidative stress induced by hypertension, and an in-depth study of their mechanism is expected to provide new targets and strategies for the prevention and treatment of HTN and related oxidative stress diseases.

## 6. Precision Antioxidant Strategies: Subcellular Modulation and Immune Integration in Hypertension Therapy

This section explores the advanced therapeutic approaches for HTN by targeting ROS through precise delivery systems and subcellular modulation, while integrating immune regulation to address HTN-related brain injuries and neurodegenerative conditions.

### 6.1. Brain-Targeted Delivery Systems for Antioxidant Therapy

ROS significantly influence subcellular dynamics, immune signaling, and neurodegenerative processes in the brain, especially in HTN [26]. Effective therapies must mitigate ROS-induced damage using brain-specific delivery systems. Nanoparticles and liposomes have emerged as promising carriers, delivering ROS scavengers directly to affected brain regions. These systems provide a controlled, sustained release of antioxidants, enhancing efficacy and reducing systemic side effects [63,64,65]. Surface modifications, like targeting ligands, improve their ability to cross the blood–brain barrier (BBB), a major hurdle in HTN where BBB integrity is often impaired [66,67,68,69,70].

Notable advancements include polymeric nanoparticles with inherent antioxidant properties that also deliver neuroprotective agents, metal-based nanoparticles like cerium oxide (CeNPs) with self-regenerating antioxidant effects, and indole derivatives with strong ROS-scavenging capabilities [71,72]. Liposomes, as lipid-based carriers, efficiently transport both hydrophilic and hydrophobic antioxidants, protecting them from degradation [70]. Receptor-mediated transcytosis further enhances nanoparticle penetration across the BBB, reducing off-target effects.

These brain-targeted systems not only combat ROS but also address inflammation, key drivers of HTN-induced brain injuries, and conditions like Alzheimer’s and Parkinson’s. Preclinical studies show they reduce cerebral ischemia-reperfusion injury, prevent cell death, and improve outcomes with lower doses, minimizing systemic risks [73]. Future research aims to refine these systems, incorporating multi-functional nanoparticles with anti-inflammatory properties for comprehensive HTN management, though long-term safety and clinical efficacy require further study [67].

### 6.2. Modulating ROS at Subcellular Levels

Targeting ROS at the subcellular level is vital for managing HTN and related chronic diseases [74,75]. Mitochondria and the endoplasmic reticulum (ER) are primary ROS sources, with mitochondria generating ROS during respiration and the ER producing ROS under stress. In HTN, mitochondrial dysfunction drives vascular and neural damage, while ER stress triggers the unfolded protein response (UPR), which, when dysregulated, increases ROS and cellular harm [76,77].

Therapies focus on selectively modulating ROS to preserve their signaling roles while preventing oxidative damage. Mitochondrial-targeted antioxidants accumulate within mitochondria, reducing excess ROS and enhancing cellular resilience without disrupting redox balance [78,79,80]. Similarly, ER-specific strategies, such as UPR pathway inhibitors, limit excessive ROS while maintaining adaptive stress responses. These approaches improve mitochondrial function, reduce damage, and support cell survival, addressing HTN’s broader impacts [81].

Emerging technologies, including mitochondrial-targeted therapies and ER stress inhibitors, reflect a nuanced understanding of ROS dynamics [82,83,84]. By balancing therapeutic efficacy with physiological ROS functions, these strategies offer a transformative foundation for treating HTN and ROS-related conditions, with potential to revolutionize disease management.

### 6.3. Targeting ROS and Inflammation in Hypertension: Bridging Subcellular Dynamics and Immune Cross-Talk

The interplay between ROS and inflammation drives neuronal injury and immune dysregulation in HTN-related brain damage, creating a feedback loop that worsens conditions like Alzheimer’s, stroke, and cognitive decline [85,86]. Effective therapies must disrupt this cycle by targeting both the ROS and inflammatory pathways [87].

Dual-targeted approaches combining ROS scavengers and anti-inflammatory agents show promise. Compounds like Ginkgolide B activate the Nrf2 pathway, boosting antioxidant defenses and reducing damage in HTN and ischemic conditions [88]. Anti-inflammatory drugs such as NSAIDs provide neuroprotection, especially when paired with ROS-targeted therapies, yielding synergistic benefits [89]. Nanotechnology enhances these efforts, with nanoparticles delivering both agents across the BBB for precise, bioavailable action [90,91,92].

The SNS also plays a role, as its heightened activity in HTN increases ROS and inflammatory cytokines, amplifying brain damage. Modulating SNS activity through bioelectronic or pharmacological means adds a complementary therapeutic layer. Future strategies will likely integrate ROS scavenging, inflammation control, and SNS regulation with personalized approaches, using genetic profiling or CRISPR-based editing to optimize outcomes.

This field underscores the importance of targeting ROS and inflammation in HTN-related brain disorders. By bridging subcellular dynamics, immune interactions, and innovative therapies, these strategies promise significant improvements for patients with hypertension-induced neurological complications [68,93].

## 7. Future Perspectives: Expanding the Research Horizon

The investigation of ROS in HTN holds significant potential for advancing scientific understanding and creating innovative treatments. Future research will likely concentrate on three interconnected domains: elucidating the influence of ROS on brain connectivity, translating discoveries from animal models to human applications, and progressing precision medicine to address ROS-related pathologies in HTN.

### 7.1. Exploring ROS and Brain Connectivity in Hypertension

ROS significantly affect brain connectivity, particularly in autonomic regions like the hypothalamus, brainstem, and prefrontal cortex, which are essential for cardiovascular regulation. In these areas, ROS impair neural communication, driving the onset and worsening of HTN. Functional connectivity studies have sought to clarify how ROS-induced neural disruptions relate to irregular blood pressure patterns, as evidenced by prior research [40,94,95].

Emerging imaging technologies, such as functional MRI (fMRI) and positron emission tomography (PET), combined with biochemical assays, enable a detailed assessment of ROS activity and its effects on neural networks. Advanced techniques, including brain-wide imaging with fluorescent probes and chemiluminescent agents, allow researchers to map ROS distribution in hypertensive models with cellular-level accuracy. These methods aim to reveal how ROS contribute to HTN-related neurodegeneration and systemic regulation, providing critical insights into the link between ROS dynamics and disease mechanisms.

### 7.2. Translating ROS Research into Human Hypertension Study

Rodent models have offered valuable insights into ROS in HTN, but applying these findings to humans is essential for clinical relevance due to notable physiological differences. To address this, advanced preclinical models, such as non-human primates and organoid systems, provide more human-like platforms to validate research outcomes.

Non-invasive imaging techniques, including magnetic resonance spectroscopy (MRS), PET, and near-infrared optical imaging, play a key role in this translational process [96]. These tools allow for the real-time observation of ROS levels and hotspots in hypertensive patients without invasive methods. Additionally, radiolabeled ROS-specific probes, such as ^18^F-labeled tracers, support longitudinal studies of ROS in the human brain [97]. Together, these advancements deepen the understanding of ROS in HTN progression and lay the groundwork for incorporating ROS-focused research into clinical trials.

## 8. Precision Medicine for ROS-Driven Hypertension

### 8.1. Translating ROS Research into Human Hypertension

Precision medicine revolutionizes the management of ROS-related HTN by customizing treatments to individual patient characteristics. Genetic studies, such as genome-wide association studies (GWAS), identify polymorphisms in ROS-regulating genes, including those encoding mitochondrial enzymes like superoxide dismutase (SOD) or components of NADPH oxidase [98,99]. These findings allow clinicians to stratify patients based on their genetic susceptibility to ROS, enabling more targeted interventions.

### 8.2. Integrating Environmental and Lifestyle Factors

Beyond genetics, environmental and lifestyle factors—such as diet, stress, and exposure to pollutants—significantly influence ROS activity. By combining genetic data with these variables, comprehensive ROS profiles can be developed. This approach supports the design of personalized interventions, including tailored antioxidant therapies and lifestyle modifications, to effectively reduce ROS-driven damage.

### 8.3. Enhancing Precision with Advanced Tools

Advanced imaging techniques and biomarker discovery further refine these personalized strategies. For example, patients with elevated ROS in specific brain regions may benefit from targeted delivery systems like nanoparticles or liposomes. These technologies deliver therapies directly to affected areas, optimizing efficacy and minimizing side effects [71,100,101]. Such innovations mark a significant leap forward in HTN treatment.

## 9. Conclusions

### 9.1. The Pivotal Role of ROS in HTN

ROS play a complex role in HTN pathophysiology, far beyond being mere metabolic byproducts. They regulate subcellular dynamics, immune responses, and neural function. In HTN, excessive ROS disrupt neural connectivity in autonomic brain regions, impair mitochondrial and endoplasmic reticulum function, and fuel a cycle of inflammation and oxidative stress. These effects drive vascular damage, neuronal injury, and cognitive decline, establishing ROS as both biomarkers and therapeutic targets.

### 9.2. Emerging Therapeutic Strategies

New therapies aim to neutralize harmful ROS effects while preserving their beneficial signaling roles. Advanced delivery systems, such as nanoparticles and liposomes, enable precise antioxidant targeting of HTN-affected brain regions. Subcellular strategies focusing on mitochondria and the endoplasmic reticulum show promise in restoring cellular balance and preventing neurodegeneration. Precision medicine, integrating genetic and environmental profiling, further personalizes these treatments by addressing individual ROS variations.

### 9.3. Challenges in Translational Research

Despite progress, translating preclinical findings—often derived from rodent models—into human applications remains challenging due to physiological differences. Advanced imaging, like MRS and PET, provides non-invasive ways to monitor brain ROS levels in patients, aiding the validation of preclinical insights. Longitudinal studies using these tools are critical to understanding ROS-driven HTN progression and evaluating therapy effectiveness.

### 9.4. Future Directions and Collaborative Efforts

Future research must focus on robust methods to assess the safety and long-term outcomes of ROS-targeted therapies across diverse populations. Collaboration among neuroscientists, cardiologists, and bioengineers is essential to refine these approaches for clinical use. Large-scale clinical trials will be key to confirming efficacy and establishing guidelines for integrating these therapies into standard HTN care.

### 9.5. A Holistic Approach to HTN Management

Tackling the interplay between ROS and HTN demands a multi-faceted strategy, integrating innovative therapies, cutting-edge imaging, and dedicated translational research. By emphasizing brain ROS in HTN pathophysiology and pursuing targeted interventions, the field is well positioned to reduce hypertension-related brain complications. This comprehensive approach promises therapies that are both effective and tailored to individual needs, heralding a new era in HTN management.

## Data Availability

This article is a narrative review synthesizing existing literature on the role of reactive oxygen species (ROS) in hypertension, with a focus on brain-targeted mechanisms, subcellular dynamics, immune cross-talk, and therapeutic pathways. No new experimental data were generated or analyzed in this study. All data and information presented are derived from publicly available sources, including peer-reviewed publications and scientific databases, as cited in the reference list. Readers are referred to the original studies cited throughout the article for access to the primary data. No additional datasets, code, or materials were produced as part of this review.
